# Association of G6PD status and haemolytic anaemia in patients receiving anti-malarial agents: a systematic review and meta-analysis

**DOI:** 10.1186/s12936-023-04493-7

**Published:** 2023-03-05

**Authors:** Erni J. Nelwan, Sharifah Shakinah, Adeline Pasaribu

**Affiliations:** 1 Division of Tropical and Infectious Disease, Internal Medicine Department, Faculty of Medicine Universitas Indonesia, Jakarta, Indonesia; 2 Division of Tropical and Infectious Disease, Internal Medicine Department, Cipto Mangunkusumo Hospital, Jakarta, Indonesia

**Keywords:** Antimalarial, G6PD deficiency, Primaquine, Malaria vivax, Malaria falciparum

## Abstract

**Background:**

Some anti-malarial drugs often cause haemolytic anaemia in glucose-6-phosphate-dehydrogenase deficiency (G6PDd) patients. This study aims to analyse the association of G6PDd and anaemia in malaria patients receiving anti-malarial drugs.

**Methods:**

A literature search was performed in major database portals. All studies searched using keywords with Medical Subject Headings (MeSH) were included, without date or language restriction. Pooled mean difference of haemoglobin and risk ratio of anaemia were analysed using RevMan.

**Results:**

Sixteen studies comprising 3474 malaria patients that included 398 (11.5%) with G6PDd were found. Mean difference of haemoglobin in G6PDd/G6PD normal (G6PDn) patients was − 0.16 g/dL (95% CI − 0.48, 0.15; I^2^ 5%, p = 0.39), regardless of the type of malaria and dose of drugs. In particular with primaquine (PQ), mean difference of haemoglobin in G6PDd/G6PDn patients with dose < 0.5 mg/kg/day was − 0.04 (95% CI − 0.35, 0.27; I^2^ 0%, p = 0.69). The risk ratio of developing anaemia in G6PDd patients was 1.02 (95% CI 0.75, 1.38; I^2^ 0%, p = 0.79).

**Conclusion:**

Single or daily standard doses of PQ (0.25 mg/kg/day) and weekly PQ (0.75 mg/kg/week) did not increase the risk of anaemia in G6PDd patients.

## Background

In 2021, according to the World Health Organization (WHO), approximately 241 million malaria cases occurred worldwide. Although there was a significant decrease of 20 million malaria cases compared to 2010, data from the last three years showed a trend of increasing incidence and relative to the 2019 data, a steep increase of fourteen million malaria cases occurred in 2021[1].

Efforts to eradicate malaria worldwide still face serious challenges in establishing an accurate diagnosis, adequate access to diagnostic tests, and accessible anti-malarial drugs. Misdiagnosis and mistreatment of malaria, in particular the dormant stage (hypnozoite) in the latency of *Plasmodium vivax* malaria*,* can lead to prolonged transmission and onset of resistance to countermeasures against both the parasite and its mosquito vector [2, 3]. The only available anti-malarial agents against latent malaria, the 8-aminoquinolines (primaquine and tafenoquine) cause potentially serious haemolytic anaemia in glucose-6-phosphate-dehydrogenase deficient (G6PDd) patients, and hence, very often imposes a critical barrier to safe access [4, 5].

In particular, for primaquine (PQ) treatment, the WHO recommends that G6PD status be assessed prior to high-dose PQ treatment (≥ 0.5 mg/kg). The recommended dose of PQ in all vivax and ovale malaria with G6PD normal (G6PDn) status is 0.25–0.5 mg/kg/day for 14 days, whereas in G6PD deficiency (G6PDd) patients, 0.75 mg/kg/week for eight weeks is recommended [6, 7]. Unfortunately, not all countries have adequate resources to screen patients for G6PD. Many physicians rationally decline to offer PQ to G6PD-unknown due to reasonable fear of causing harm. However, the decision likely leads to patients suffering multiple acute attacks with morbidity, risk of mortality, and onward transmission [3].

Many studies have been reported on G6PDd, anti-malarial drugs, and haemolytic anaemia. Two previous systematic reviews showed an increased risk of anaemia in G6PDd patients treated with PQ. However, the overall population was not specifically malaria patients [8, 9]. Based on previous evidence, we conducted this systematic review to evaluate the association of G6PDd and anaemia in malaria patients receiving anti-malarial drugs. These findings help inform the decisions of physicians and policymakers on G6PD screening in malaria-endemic areas.

## Methods

This systematic review and meta-analysis were conducted based on The Preferred Reporting Items for Systematic Review and Meta-Analyses (PRISMA) guidelines. The protocol of this review was registered at the Prospective Register of Systematic Reviews (PROSPERO) with code number CRD42021227813.

### Searching strategy

The literature search was conducted in July 2021 using six databases: PubMed/ Medline®, Embase®, Cochrane Central Register of Controlled Trials (CENTRAL®), EBSCOhost/CINAHL®, ProQuest®, and Scopus®, along with snowballing method from study references and hand-searching from the electronic university library and local journals or databases. All observational and experimental studies were included in this review. The keywords that were used included “malaria,” “glucose-6-phosphate-dehydrogenase/ G6PD”, “antimalarial” (including the names of antimalarial drugs used by the patients: primaquine, tafenoquine, amodiaquine, pyrimethamine, proguanil, atovaquone, artemisinin, lumefantrine, doxycycline, clindamycin), “anemia”, “hemoglobin”, and “hemolysis”, with MeSH terms in English without time of study or language restriction. Grey literature, such as abstracts, proceedings, and academic thesis, were included in the literature search. The list of keywords from respective databases is shown in Table 1.

**Table 1:** Searching for articles in databases

Source	Keywords	Hits
PubMed®	(((((((((((((antimalarial[MeSH Terms]) OR (primaquine[MeSH Terms])) OR (chloroquine[MeSH Terms])) OR (tafenoquine[MeSH Terms])) OR (amodiaquine[MeSH Terms])) OR (pyrimethamine[MeSH Terms])) OR (proquanil[MeSH Terms])) OR (atovaquone[MeSH Terms])) OR (artemisinin[MeSH Terms])) OR (lumefantrine[MeSH Terms])) OR (doxycycline[MeSH Terms])) OR (clindamycin[MeSH Terms])) AND (G6PD[MeSH Terms])) AND (anemia[MeSH Terms])	355
Scopus®	(antimalarial OR anti-malarial) AND g6pd AND (anemia OR anaemia OR hemoglobin OR haemoglobin) AND (LIMIT-TO (DOCTYPE, *“ar”*)) AND (LIMIT-TO (OA, *“all”*))	388
ProQuest®	ab(antimalarial OR anti*malarial) AND ab(g6pd) AND ab(anemia OR anaemia OR hemoglobin OR haemoglobin)	18
EBSCOhost/ CINAHL®	AB ( antimalarial OR primaquine OR chloroquine OR tafenoquine OR amodiaquine OR pyrimethamine OR proguanil OR atovaquone OR artemisinin OR lumefantrine OR doxycycline OR clindamycin) AND AB G6PD AND AB ( anaemia OR anemia OR hemoglobin OR haemoglobin)	136
CENTRAL®	(antimalarial OR anti*malarial):ti,ab,kw AND (G6PD):ti,ab,kw AND (anemia OR anaemia OR hemoglobin OR haemoglobin):ti,ab,kw	32
Embase®	antimalarial:ti,ab,kw AND g6pd:ti,ab,kw AND anemia:ti,ab,kw	653

CENTRAL Cochrane Central Register of Controlled Trials

CINAHL Cumulative Index to Nursing and Allied Health Literature

ti,ab,kw: title, abstract, keyword

### Inclusion and exclusion criteria

Studies included in this systematic review and meta-analysis had to meet the following criteria: (1) adult male or female patients with confirmed malaria infection (*Plasmodium falciparum, Plasmodium vivax*, and/or mixed infection); (2) treatment with anti-malarial agents known to cause haemolysis occurred; and (3) patients underwent evaluation of G6PD status, anaemia, and/or haemolysis evaluation. Study subjects with only paediatrics and/or normal G6PD level were excluded from the systematic review. No animal, in-vitro, or healthy volunteer studies were included.

Malaria diagnosis and species of infection were defined by positive thin and thick blood film examination by microscopy on day 0. Definition of G6PD deficiency, anaemia and/or haemolysis, and haemoglobin level were determined based on standard examination in respective studies. The use of anti-malarial agents included drugs such as primaquine, tafenoquine, amodiaquine, pyrimethamine, proguanil, atovaquone, artemisinin, lumefantrine, doxycycline, and clindamycin.

### Data extraction

Literature search was conducted by AP, whereas both title/abstract screening and full-text reading were reviewed by two independent investigators (SS, AP), with the help of Covidence® online software. Any disagreements during the process were discussed and resolved by a third independent investigator (EJN).

Data extraction comprised of author’s name, year of study, country, study drugs and dose, number of participants, gender, mean age, type of malaria infection, method of study, and duration of follow-up. The outcomes of the study were anaemia, level of haemoglobin, or haemolysis status, depicted in the mean difference of haemoglobin or risk ratio of anaemia. When unavailable, data on the outcomes were described in the text review.

### Quality assessment

Selected studies were critically reviewed based on the type of studies. Randomized controlled trials (RCTs) were assessed using Cochrane risk-of-bias tool for randomized trials (RoB 2)[10], and non-randomized trials were assessed with Risk of Bias in Non-Randomized Studies—of Interventions (ROBINS-I)[11], whereas observational cohort studies were assessed with Newcastle–Ottawa Scale (NOS) checklist for cohort studies [12]. RoB 2 quality was classified into low, with some concerns, or high risk of bias; ROBINS-I was classified into low, moderate, serious, critical risk of bias, or no information; and NOS for cohort studies was classified into high-quality study, high risk of bias, and very high risk of bias studies. When the data to assess the risk of bias was unclear, attempts were made to contact the authors for clarification. The quality of evidence was assessed by using the Grading of Recommendations Assessment, Development and Evaluation (GRADE) approach [13].

### Statistical analysis

Statistical analysis was performed using RevMan® version 5.4.1. Data were presented in tables, and the results of the analysis were presented in the forest plot (when possible) or text review. In the absence of statistical heterogeneity, a fixed-effects model was used; otherwise, a random-effects model was performed in more heterogeneous trials. When available, the values were reported as mean differences in haemoglobin level and risk ratio of anaemia as aggregated data, with a 95% confidence interval (95% CI) and p-value. Heterogeneity was identified using the I^2^ statistic, in which value I^2^ 0%-40%: might not be important, 30%-60%: moderate heterogeneity, 50%-90% substantial heterogeneity, and 75%-100%: considerable heterogeneity.

## Results

**Fig. 1:**
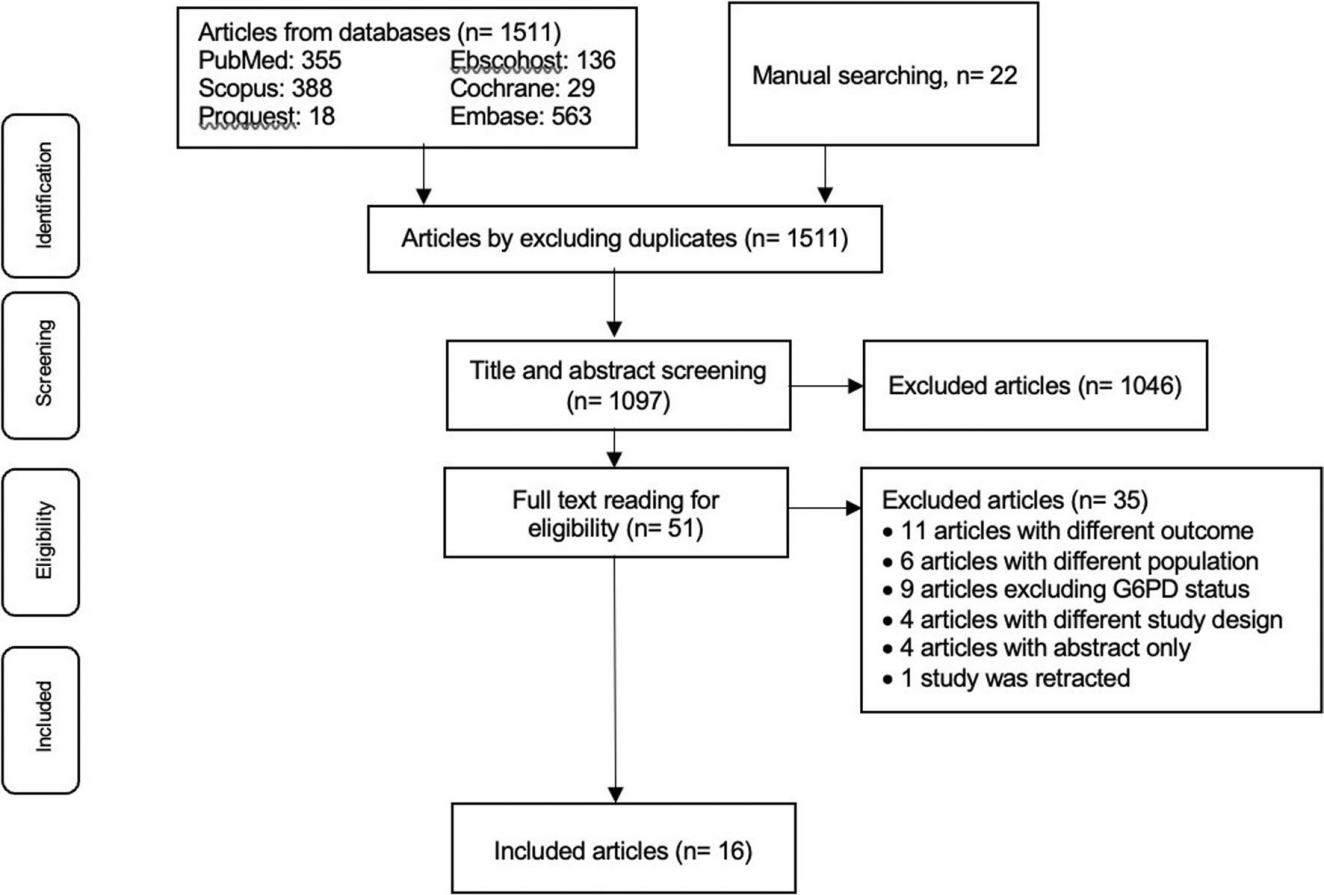
Study selection flow diagram

The initial literature search found 1533 articles from databases and manual searching. After excluding duplicates, 1097 articles were screened based on their title and abstract. A total of 1046 articles were excluded, leaving 51 articles for full-text reading and assessment of eligible articles. Thirty-five studies were excluded, as depicted in Fig. 1. Eleven articles did not include haemoglobin level and/or anaemia and/or haemolysis status as an outcome; one study was done only on the paediatric population; 5 studies were done on healthy subjects, using anti-malarials as prophylaxis; 9 studies excluded G6PDd patients; 2 studies were preliminary studies, and 2 studies were cross-sectional studies. In summary, 16 articles were included in this systematic review. The publication year for the articles ranged from 1981 to 2021, most were published in English, and one article was in Portuguese.

Sixteen selected articles comprised 3,415 malaria patients treated with studied anti-malarial agents, and 400 (11.7%) had G6PDd. Seven articles reported only malaria caused by *P. falciparum,* five articles reported only malaria caused by *P. vivax,* and the rest studied malaria caused by both parasites and/or mixed infection [14–18]. Searching was done for anti-malarials not limited only to PQ, but most studies (14 articles) [14–17, 19–29] reported the study of PQ, and the two remaining studies [18, 20] reported the use of other anti-malarials: chlorproguanil–dapsone–artesunate (CDA), chlorproguanil–dapsone (CPG–DDS), sulfadoxine-pyrimethamine (SP), and chloroquine (CQ). However, the study population with CDA, CPG-DDS, SP, and CQ only was not included in the meta-analysis due to the limited study. There was only one study for each drug; therefore the meta-analysis for non-PQ drugs could not be performed. The dose of PQ used in each article was various. Some of the studies used more than one regimen dose for PQ; four studies used a single PQ dose of 0.25 mg/kg, one study used a single 15 mg PQ, one study used a single PQ dose of 0.25–0.5 mg/kg, one study used a single PQ dose of 0.75 mg/kg, three studies used daily 0.25 mg/kg/day PQ for fourteen days, three studies used daily 0.25–0.5 mg/kg PQ for seven to fourteen days, one study used daily 15 mg PQ for fourteen days, one study used PQ dose of ≥ 0.5 mg/kg/day for fourteen days, and two studies used PQ 0.75 mg/kg/weekly for 8 weeks. The duration of treatment follow-up also varied, ranged from 7 to 30 days, depending on the species involved.

All selected articles reported outcomes of anaemia or haemolysis, but only one half of them reported the outcomes as mean difference of haemoglobin level between G6PDn and G6PDd patients, risk ratio of anaemia, or both, and hence, not all articles could be further analysed in the meta-analysis [19–22, 24, 25, 27, 28]. The outcomes of the other one half portion of the selected articles were presented as mean or median haemoglobin level, haemolysis incident, rate of anaemia, or mean percentage of fractional haemoglobin reduction [14–18, 23, 26, 29]. G6PD deficiency was established through various methods, such as by biochemical phenotyping examination (qualitative or quantitative) followed by genotyping assessment, phenotyping or genotyping examination only, or even not mentioned in the article. The summary of articles is shown in Table 2.

**Table 2:** Summary of characteristics of study participants

No	Author	Country	Number of Subjects^a^	Age range	Type of Malaria	Method	Studied drugs, doses (duration)	Duration of Follow Up	Mean difference (SD or 95% CI), G6PD def. vs. normal	Risk ratio anaemia (95% CI)	Other related outcomes
Single dose 0.25 mg/kg PQ											
1	Mwaiswelo et al. [24]	Tanzania	88 G6PDn, 21 G6PDd	≥ 1 y.o	*P.f*	RCT	PQ 0.25 mg/kg (SD)	7 days	1.16 (0.67–1.66) vs. 0.81 (0.59–1.02)	N/A	Mean relative Hb reduction (%) (95% CI) G6PD def. vs. normal: 7.9 (4.58–11.18) vs. 7.2 (5.04–9.35)
2	Bastiaens et al. [19]	Burkina Faso, Gambia	20 G6PDn, 42 G6PDd	10–45 y.o	*P.f*	RCT	1. PQ 0.25 mg/kg (SD)	7, 28 days	**Burkina Faso** Day 7, dose 0.25 mg/kg: − 0.92 (1.43) vs − 0.64 (1.01) Day 28, dose 0.25 mg/kg: − 0.29 (1.83) vs 0.04 (1.27) **Gambia** Day 7, dose 0.25 mg/kg: − 0.99 (0.85) vs − 1.06 (1.55) Day 28, dose 0.25 mg/kg: − 0.52 (1.18) vs − 0.4 (2.01)	Changes in Hb conc. > − 2.5 g/dL: **Burkina Faso** Dose PQ 0.25 mg/kg RR = 1.13 [0.46, 2.77] **Gambia** Dose PQ 0.25 mg/kg RR = 0.60 [0.17, 2.14]	N/A
3	Dysoley et al. [21]	Cambodia	47 G6PDn, 9 G6PDd	4–76 y.o	*P.f*	RCT	PQ 0.25 mg/kg (SD)	7 days	− 1.46 (− 3.3–0.4) g/dL vs. − 1.24 (− 4.6–2.4) g/dL	Hb reduction ≥ 25% normal, RR: 1.04 [0.14, 7.91]	Mean Hb, G6PD def. vs. normal: 10.9 (8.5–13.2) vs. 12.05 (8.7–14.5)
4	Raman et al. [23]	South Africa	55 G6PDn, 11 G6PDd	≥ 2 y.o	*P.f*	RCT	PQ 0.25 mg/kg (SD)	7, 14, 28, 42 days	N/A	N/A	11 subjects with G6PD deficiency reached the lowest median Hb during day 7, compared to G6PD normal group
Single dose 15 mg											
1	Tine et al. [22]	Senegal	105 G6PDn, 30 G6PDd	18–74 y.o	*P.f*	RCT	PQ 15 mg (SD)	7, 28 days	Day 7, G6PD def. vs normal: − 1.75 (− 6.1, 2.6) vs − 1.36 (− 4.7, 3.3) Day 28, G6PD def. vs normal: − 0.24 (− 3.3, + 3) vs – 0.32 (− 3.7, + 4.8)	Hb reduction > 2 g/dL in day 7, RR = 1.12 [0.78, 1.62]	Mean Hb, Day 28, G6PD def. vs. normal: 12,8 (0.87) vs. 13,2 (1.26)
Single dose 0.25–0.5 mg/kg											
1	Bastiaens GJH et al. [19]	Burkina Faso, Gambia	11 G6PDn, 24 G6PDd	10–45 y.o	*P.f*	RCT	PQ 0.4 mg/kg (SD)	7, 28 days	**Burkina Faso** Day 7, dose 0.4 mg/kg: − 0.93 (1.20) vs − 0.60 (1.70) Day 28, dose 0.4 mg/kg: − 0.10 (1.13) vs − 0.12 (2.01)	Changes in Hb conc. > − 2.5 g/dL: Dose PQ 0.4 mg/kg RR = 0.70 [0.30, 1.66]	N/A
Single dose 0.75 mg/kg											
1	Ley et al. [29]	Bangladesh	91 G6PDn, 4 G6PDd	> 1 y.o	*P.f*	Cohort	PQ 0.75 mg/kg, SD (*P.f*)	30 days	N/A	N/A	Mean fractional Hb reduction in day 7, G6PD def. vs normal: − 9.1% (− 53.2%, + 35.0%) vs. + 2.9% (− 1.14%, + 6.8%), p = 0.739
PQ daily ≤ 0.25 mg/kgb											
1	Poirot et al. [25]	Senegal, Bangladesh, Tanzania, Swaziland	107 G6PDn, 16 G6PDd	> 1 y.o	*P.f, P.v,* mix	RCT & cohort	PQ ≤ 0.25 mg/kg	7 days	PQ ≤ 0.25 mg/kg: − 1.38 [− 2.13, − 0.63] vs. − 1.27 [− 1.56, − 0.99]	N/A	N/A
2	Avalos S et al. [28]	Honduras	48 G6PDn, 7 G6PDd	> 10 y.o	*P.f, P.v*	Cohort	PQ 0.25 mg/kg, 14d in *P. vivax* PQ 0.75/kg, SD in *P. falciparum*	7 days	All doses: − 1.63 (1.79) vs. − 0.28 (1.18), p = 0.040	N/A	Mean Hb, G6PD def. vs. G6PD normal: 9.88 (1.63) vs. 12.07 (1.4), p = 0.040
3	Leslie et al. [14]	Pakistan	121 G6PDn, 1 G6PDd	N/A	*P.v*	RCT	1. PQ 0.25 mg/kg (14 days) 2. PQ 0.75 mg/kg/week (8 weeks)	14 days	N/A	N/A	On day 7, mean Hb *fell* below mean Hb in G6PD normal: 10.0 g/dL vs 12.6 g/dL), and *rise back* to normal in day 14: 12.7 g/dL vs. 12.6 g/dL)
PQ daily > 0.25–0.5 mg/kgb											
1	Brito− Sousa JD et al. [17]	Brazil	672 G6PDn, 94 G6PDd	4–84 y.o	*P.v*	Cohort	PQ 0.5 mg/kg, 7 days	7 days	N/A	N/A	Rate of severe anemia in G6PD def. was 55/94 or 58.5% (no comparison to G6PD normal group)
2	Silva et al. [16]	Brazil	8 G6PDn, 3 G6PDd	18–60 y.o	*P.v*	Cohort	PQ 0.5 mg/kg (7 days)	3 days	N/A	N/A	Mean Hb, G6PD def. vs. normal: 7.8 ± 0.3 vs. 12.73 ± 1.38
3	A. Llanos-Cuentas et al. [15]	Peru, Brazil, Colombia, Vietnam, Thailand	85 G6PDn & G6PDd, 6 G6PDd in all group studies	> 16 y.o	*P.v*	RCT	PQ 15 mg (14 days))	14 days	N/A	N/A	Among 5 subjects with protocol-defined Hb decreases, none are G6PD def. patient
4	Khoo KK et al. [26]	Malaysia	23 G6PDd	> 1 y.o	*P.f, P.v,* mix	non-RCT	CQ + PQ – PQ 75 mg (3 days) (*P.f*) – PQ 210 mg (14 days) (*P.v,* mix)	3, 14 days	N/A	N/A	Hemolysis in CQ + PQ group: 7/23 (30.4%)
5	Poirot et al. [25]	Senegal, Bangladesh, Tanzania, Swaziland	125 G6PDn, 15 G6PDd	> 1 y.o	*P.f, P.v,* mix	RCT & cohort	1. PQ 0.25–0.5/kg	7 days	PQ > 0.25—≤ 0.5 mg/kg: − 1.19 [− 1.97, − 0.42] vs. − 0.63 [− 0.90, − 0.37]	N/A	N/A
PQ daily > 0.5 mg/kg											
1	Poirot et al. [25]	Senegal, Bangladesh, Tanzania, Swaziland	152 G6PDn, 4 G6PDd	> 1 y.o	*P.f, P.v,* mix	RCT & cohort	2. PQ > 0.5 mg/kg (SD or 14 days)	7 days	PQ > 0.5 mg/kg: − 2.75 [− 4.22, − 1.28] vs. − 0.86 [− 1.1, − 0.62]	N/A	N/A
PQ 0.75 mg/kg weekly											
1	Kheng et al. [27]	Cambodia	57 G6PDn, 18 G6PDd	> 1 y.o	*P.v,* mix	non-RCT	PQ 0.75 mg/kg/week (8 weeks)	7, 28 days	D7: − 2.2 (− 4.9, 0.8) vs. − 0.5 (− 2.2, 2.8) D28: 0.3 (− 2.3, 2.7) vs. 0.2 (− 2, 2.6)	Hb reduction > 25% on day 7, RR: 33.58 [1.95, 579.53]	Mean Hb, G6PD def. vs. normal: D7: 10.9 (8.9, 13) vs. 13 (9.5, 15.7) D28: 13.5 (9.5–16.1) vs. 12.8 (10.9–15.4)
CPG-DDS											
1	Leslie et al. [18]	Afghanistan, Pakistan	300 G6PDn,2 G6PDd	> 3 y.o	*P.v*	RCT	CPG-DDS (3 days)	14	N/A	N/A	1 subject with G6PDd had Hb decreased 1.4 g/dL over 24 h
2	Tiono et al. [20]	Burkina Faso, Ghana, Mali, Nigeria	824 G6PDn, 68 G6PDd	> 1 y.o	*P.f*	RCT	1. CDA 2. CPG-DDS	28 days	N/A	Hb reduction ≥ 4 g/dL or ≥ 40% baseline of Hb 5 g/dL or blood transfusion: – CDA, RR = 33.09 [11.27, 97.15] – CPG-DDS, RR = 10.74 [3.93, 29.37]	N/A
Tafenoquine											
1	A. Llanos- Cuentas et al.)[15]	Peru, Brazil, Colombia, Vietnam, Thailand	166 G6PDn & G6PDd, 6 G6PDd in all group studies	> 16 y.o	*P.v*	RCT	Tafenoquine 300 mg (SD)	14 days	N/A	N/A	Among 5 subjects with protocol-defined Hb decreases, none are G6PD def. patient

^a^Number of study subjects receiving study drugs

PQ: primaquine; SD: single dose; *P.f*: *Plasmodium falciparum*; *P.v*: *Plasmodium vivax*; N: number of study participants using the study drug/s; CPG-DDS: chlorproguanil-dapsone; CDA: chlorproguanil-dapsone-artesunate; G6PDd: glucose-6-phosphate dehydrogenase deficiency; G6PDn: glucose-6-phosphate dehydrogenase normal; N/A: data not available; RR: risk ratio; RCT: randomized controlled trial

### Risk of bias assessment

The selected RCTs were evaluated for risk of bias with ROB 2 and showed some concerns, low, and high risk of bias (Table 3). Studies with high risk of bias indicated concerns in missing outcome data, deviation from intended intervention, or process of randomization. Among non-RCTs, only three studies presented as high quality, whereas others had moderate or high risk of bias, mainly in the area of identification of participants and assessment of the outcomes [25, 28, 29]. Complete risk of bias assessment figures were depicted in Tables 3, 4, and 5.

**Table 3:** Risk of bias of randomized controlled trial studies with ROB 2

Study	Randomization process	Deviation from intended interventions	Missing outcome data	Measurement of the outcome	Selection of reported result	Overall bias
Bastiaens et al. [19]	+ +	+	+ + +	+	+	Some concerns
Tiono et al. [20]	+	+	+ + +	+	+	Low risk
Dysoley et al. [21]	+	+ +	+ + +	+	+	High risk
Tine et al. [22]	+	+	+	+	+	Low risk
Raman et al. [23]	+	+ +	+ + +	+	+	Low risk
Leslie et al. [18]	+	+ + +	+	+	+	High risk
Llanos-Cuentas et al. [15]	+	+	+	+	+	Low risk
Mwaiswelo et al. [24]	+	+	+	+	+	Low risk
Leslie et al. [14]	+ + +	+	+	+	+	High risk

+ : low risk, +  + : some concerns, +  +  + : high risk

*ROB 2* Cochrane risk-of-bias tool for randomized trials

**Table 4:** Risk of bias of non-randomized studies for intervention with ROBINS-I

Study	Confounding	Selection of participants	Classification of interventions	Deviations from intended interventions	Missing data	Measurement of outcomes	Selection of reported result	Overall
Khoo et al. [26]	+ + +	+	+ + +	+ +	~	+ +	+ +	Serious risk
Kheng et al. [27]	+ +	+	+	+ +	+	+	+	Moderate risk

+ : low risk, +  + : moderate risk, +  +  + : serious risk, +  +  +  + : critical risk, ~ : no information

*ROBINS I* Risk of Bias in Non-Randomized Studies-of Interventions

**Table 5:** Risk of bias of cohort studies with NOS

Study	Selection				Comparability	Outcome			Total	Quality
	Representativeness	Selection of non-exposed cohort	Ascertainment of exposure	Outcome of interest at the start of the study		Assessment of outcome	Duration of follow up	Adequacy of follow up cohorts		
Poirot et al. [24]	*	*	*	*	*	*	*	*	8	High quality
Silva et al. [15]		*	*			*			3	Very high risk of bias
Avalos S et al. [27]	*	*	*	*	*	*	*	*	8	High quality
Brito-Sousa JD et al. [16]	*	*	*			*	*		5	High risk of bias
Ley et al. [28]	*	*	*	*	*	*	*	*	8	High quality

*Certainty of Evidence*

Our assessment of the quality of evidence using the GRADE approach is presented in the summary of findings in Table 6

*NOS* Newcastle–Ottawa Scale

### Mean difference of haemoglobin level

**Fig. 2:**
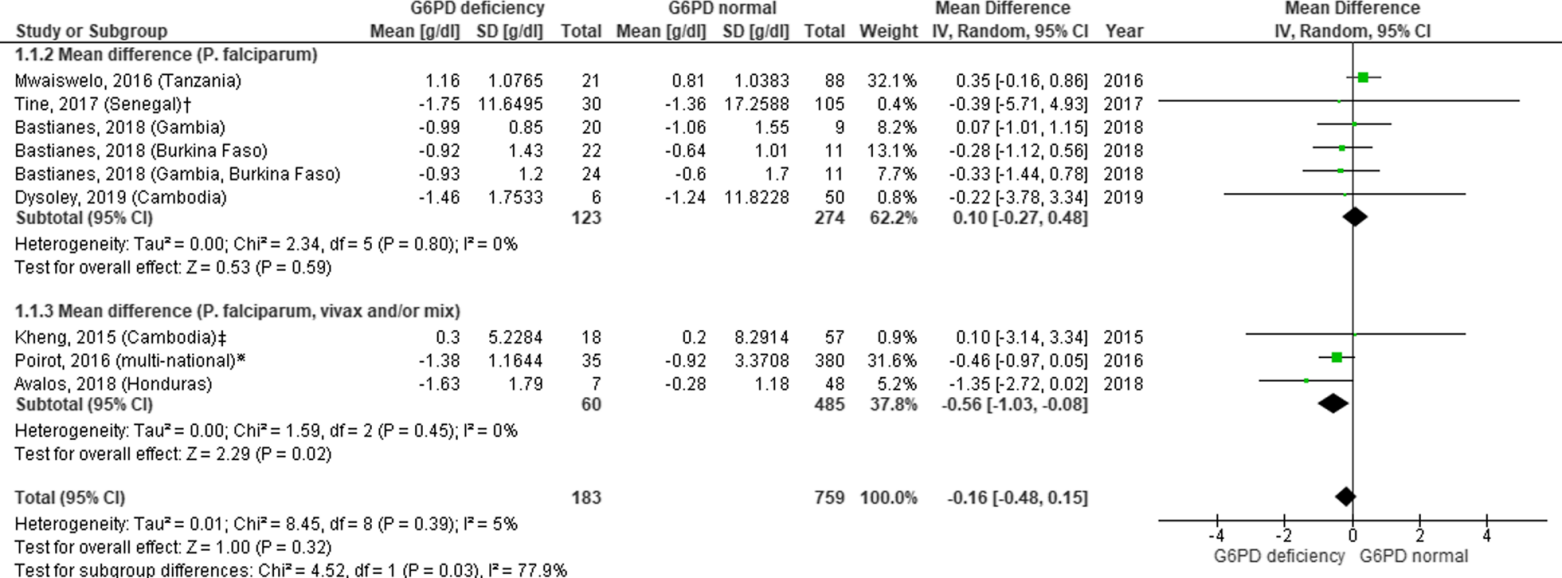
Mean difference of haemoglobin level in G6PD deficient and G6PD normal patients receiving primaquine based on the type of malaria. Data of mean difference was presented as g/dL, standard error (SE), and 95% confidence interval of mean difference. Differences in haemoglobin level were measured between day 0 (baseline) and day 7 or 28, depending on the type of infection and study design. Doses of primaquine vary. *SE* standard error, *G6PD* Glucose-6-phosphate-dehydrogenase; ^†^analysis of study within 7 days of observation; ‡analysis of study within 28 days of observation; *analysis of study with all doses of primaquine included in the study

Data of mean difference of haemoglobin level before and after anti-malarials administration in G6PDd and G6PDn patients were only available in seven studies comprising nine groups that could be further analysed in the forest plot. The mean difference of haemoglobin in G6PDd patients is higher than the G6PDn patients [− 0.70 (95% CI − 1.91, 0.50) vs. 0.27 (95% CI 0.09, 0.45)]. When both groups were further analysed, the gap of mean difference between G6PDd and G6PDn group was lower in the G6PDd group -0.16 (95% CI − 0.48, 0.15); however, it was not statistically nor clinically significant (Fig. 2). When analysed separately according to whether the infection was caused by *P. falciparum* only or *P. vivax* and/or mixed infection, the mean difference of haemoglobin level showed a slight gap between each group [0.1 (95% CI − 0.27, 0.48) and − 0.56 (95% CI − 1.03, − 0.08)].

**Fig. 3:**
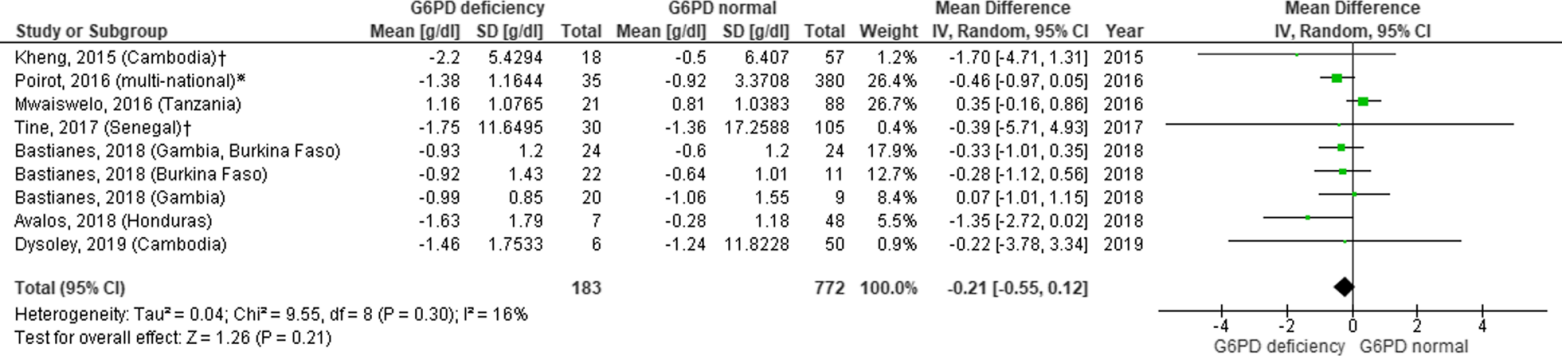
Mean difference of haemoglobin in G6PD deficient and G6PD normal patients receiving primaquine with a duration of follow-up of seven days (D0 and D7). Data of mean difference was presented as g/dL, standard error (SE), and 95% confidence interval of mean difference. Doses of primaquine vary. *SE* standard error, *G6PD* Glucose-6-phosphate-dehydrogenase; ^†^analysis of study within 7 days of observation; *analysis of study with all doses of primaquine included in the study

**Fig. 4:**
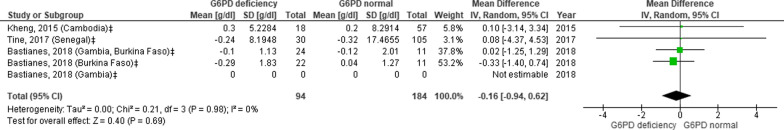
Mean difference of haemoglobin in G6PD deficient and G6PD normal patients receiving primaquine with a duration of follow-up of 28 days (D0 and D28). Data of mean difference was presented as g/dL, standard error (SE), and 95% confidence interval of mean difference. Doses of primaquine vary. *SE* standard error, *G6PD* Glucose-6-phosphate-dehydrogenase; ‡analysis of study within 28 days of observation

The analysis of seven articles (nine study groups) in Fig. 2 were performed with variance in drug doses and duration of follow-up. Analysis of the mean difference in haemoglobin level in the respective two aspects was done. Both groups, with duration of follow-up of seven days or 28 days, showed a slight gap in mean difference, which was not statistically nor clinically significant [0.21 (95% CI − 0.51, 0.09) and − 0.16 (95% CI-0.94, 0.62)] (Figs. 3 and 4).

**Fig. 5:**
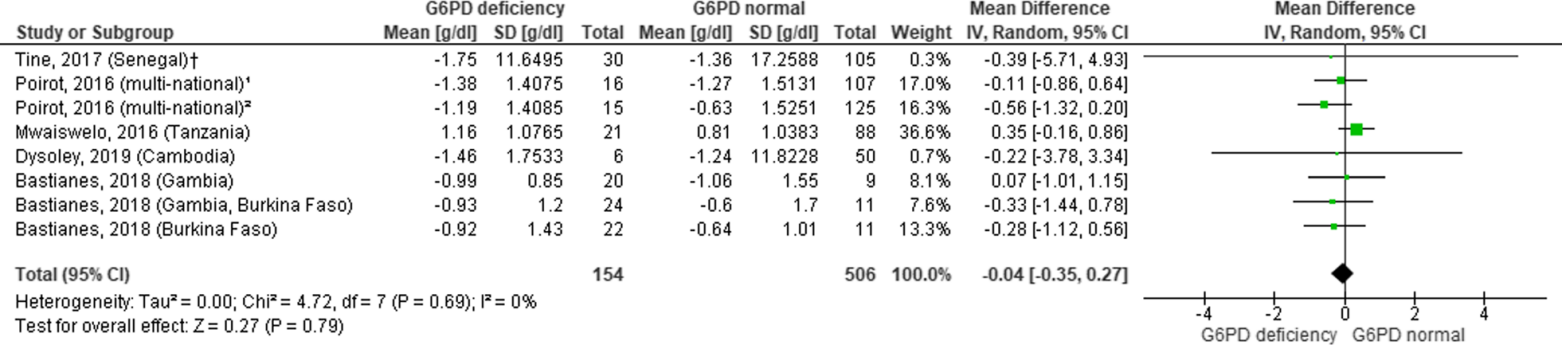
Mean difference of haemoglobin in G6PD deficient and G6PD normal patients with administration of low primaquine dose < 0.5 mg/kg/day. Data of mean difference was presented as g/dL, standard error (SE), and 95% confidence interval of mean difference. Doses of primaquine vary. *SE* standard error, *G6PD* Glucose-6-phosphate-dehydrogenase, *PQ* primaquine; †analysis of study within 7 days of observation; ^1^analysis of study with primaquine dose ≤0.25 mg/kg/day; ^2^analysis of study with primaquine dose 0.25–0.5 mg/kg/day

Figure 5 showed a forest plot of mean difference on studies with administration of PQ dose < 0.5 mg between G6PDd and G6PDn group with value − 0.04 (95% CI − 0.35, 0.27). This value was lower compared to the result by Poirot [25] who studied a group with PQ dose administration > 0.5 mg/kg/day in G6PDd and G6PDn patients [mean difference − 2.75 (95% CI − 4.22, − 1.28) and − 0.86 (95% CI − 1.1, − 0.62), respectively].

### The risk of developing anaemia after anti-malarial therapy

**Fig. 6:**
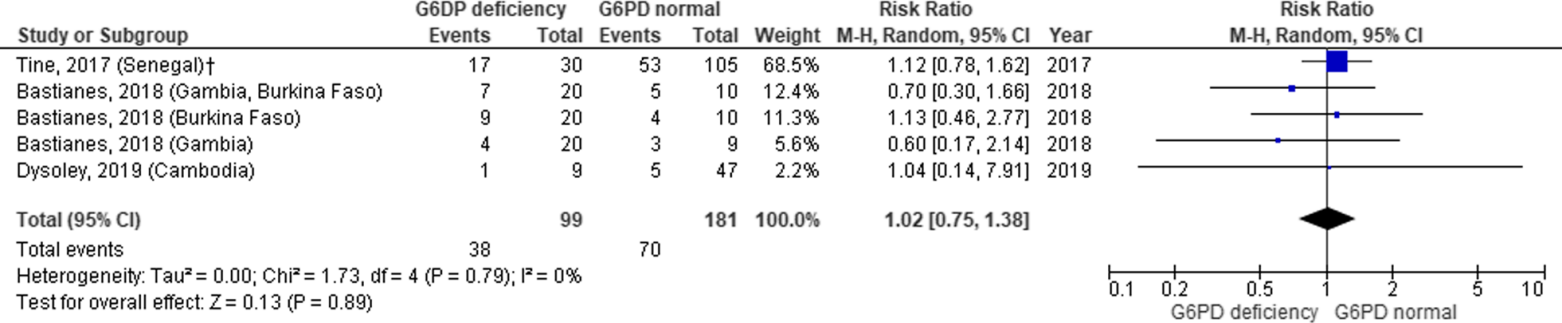
Risk ratio of developing anaemia in G6PD deficiency and G6PD normal before and after single dose or daily primaquine treatment. Data of mean difference was presented as g/dL, standard error (SE), and 95% confidence interval of mean difference. Doses of primaquine vary. *SE* standard error, *G6PD* Glucose-6-phosphate-dehydrogenase; ^†^analysis of study within 7 days of observation

Three studies [19–22, 27] evaluated the risk of developing anaemia after a single dose or a daily dose of PQ in the G6PDd and G6PDn group with an overall risk ratio of 1.02 (95% CI 0.75, 1.38) (Fig. 6).

## Discussion

In the study protocol, many anti-malarials were included to evaluate their effect on the G6PDd population, however, most of the studies were done using primaquine and only a small number of studies used other anti-malarials, such as tafenoquine, chlorproguanil–dapsone–artesunate (CDA), chlorproguanil–dapsone (CPG–DDS), sulfadoxine-pyrimethamine (SP), chloroquine; therefore, we only examined the pooled effect of primaquine. 16 studies on malaria patients treated with anti-malarial agents were analyzed. Among 3,474 patients, 11.5% of them are G6PD deficient. Most studies evaluated PQ as the investigational drug and excluded severe malaria infection. PQ is one of the drugs to be wary of due to the high risk of haemolytic anaemia [4, 30].

Regardless of the type of *Plasmodium* species and the administered anti-malarial drugs, forest plot of seven studies showed a small mean difference of haemoglobin level between G6PDd and G6PDn group (− 0.16 g/dL) (Fig. 2). Other studies not included in the meta-analysis had similar outcomes. One study with PQ in South Africa [23] measured the median haemoglobin level and found that G6PDd subjects reached a lower haemoglobin level compared to G6PDn subjects, however, its nadir point was 11.5 g/dL and it was clinically harmless. Other studies in single low dose PQ in *P. falciparum* infection also highlighted no difference in mean haemoglobin level or percentage of mean relative haemoglobin reduction between G6PDd and G6PDn subjects [22, 24].

A study by Llanos-Cuentas compared the haemolytic effect of primaquine and tafenoquine. The results showed that the decrease in haemoglobin levels occurred in the normal G6PD group and did not occur in the G6PDd group, although the number of subjects in the G6PDd group was very low. Decreased haemoglobin levels occurred in the tafenoquine and primaquine groups but were self-limited without requiring special treatment [15].

Based on these studies, it was presumed that anti-malarial treatment in non-severe malaria patients would be unlikely to cause a clinically significant side effect of anaemia. Analysis of three studies on PQ also did not show an increased risk ratio of developing anaemia in G6PDd patients, hence, confirming the safety of PQ (Fig. 6).

Almost half of the articles included in this review were performed in *P. falciparum* infection only [19–24, 29]. Based on the subgroup analysis of forest plot, the mean difference of haemoglobin level between *P. falciparum* and *P. vivax* and/or mixed infection was not statistically nor clinically significant. Other studies on *P. vivax* that were not included in the forest plot showed various outcomes [15–18, 26]. A multinational RCT with low dose PQ (15 mg) and single-dose tafenoquine observed no significant decrease of haemoglobin level in the G6PDd group [15]. In contrast, one cohort in Brazil with higher dose PQ (0.5 mg/kg/day, seven days) displayed a lower mean of haemoglobin level in G6PDd group [16]. However, this study had small number of study subjects and a relatively short duration of follow-up (3 days), therefore, further higher quality study is needed. Two other studies in *P. vivax* and/or mixed infection revealed an increased risk of haemolysis and severe anaemia in PQ administration, nevertheless, they did not perform a comparison to the G6PDn group [17, 26].

Instead of the type of the *Plasmodium* species, one presumably important condition that might add the risk of anaemia was the accumulation of administered dose of PQ. When PQ was given in a standard low dose (≤ 0.5 mg/kg/day), the result of the forest plot for the mean difference of haemoglobin level between G6PDd and G6PDn groups was not statistically nor clinically significant. Results from other studies with administration of PQ dose < 0.5 mg/kg/day) (not included in the forest plot) also disclosed a consistent outcome [15, 23]. When given a higher dose (0.5 mg/kg/day) of PQ, subjects showed a significant decrease in mean haemoglobin level and a high rate of severe anaemia in G6PDd patients up to 50% [16, 17]. Studies with PQ dose > 0.5 mg/kg/day in G6PDd patients also showed a significant fall in haemoglobin level and an increased risk of haemolysis up to 30% [25, 26]. There were two studies that used 0.75 mg/kg/week of PQ for 8 weeks. Both studies showed a decline in mean haemoglobin level during the first week, which eventually went up to a normal level in the third and fourth week of drug treatment. The author concluded that the administration of a low dose of PQ (< 0.5 mg/kg/day) and a weekly dose of PQ (0.75 mg/kg/week for eight weeks) in G6PDd patients did not increase the risk of anaemia.

The change of mean difference of haemoglobin level also showed no significant decrease during seven or 28-days follow-up period, indicating that anaemia as a side effect in the administration of anti-malarial was not hazardous in both short and long-term evaluation. Haemolytic anaemia caused by G6PDd is usually a self-limited condition, and anaemia is usually resolved when the precipitating drugs are discontinued.

Among sixteen studies in this review, half were classified as low risk of bias. Some pooled analyses showed high heterogeneity and, therefore, subgroup analysis was performed which exhibited significant results and lower heterogeneity. The funnel plot could not be arranged because of the lack of studies in meta-analysis.

This review is the first systematic review of the effect of G6PD status, which includes only malaria patients receiving anti-malarial agents. A particular strength of this study is that all of the included study was high-quality cohort and randomized controlled trials with before and after endpoints, thus, the authors are confident in this study’s ability to demonstrate a causal relationship between drug administration and haemoglobin level. In the table displaying the summary of findings (Table 6), the certainty of the evidence was downgraded one level due to the serious risk of inconsistency and imprecision. Based on GRADE evaluation, the outcome of this study showed a high and moderate certainty of evidence. Currently, no publication bias was suspected for the outcome included in this review.

**Table 6:** Summary of the findings

Association of G6PD status and haemolytic anaemia in patients receiving anti-malarial agents: a systematic review and meta-analysis					
**Patient or population:** people with malaria infection receiving anti-malarial agents **Intervention:** G6PD deficiency (G6PDd) **Comparison:** G6PD normal (G6PDn)					
Outcome	No. of Participants (studies)	Quality of the evidence (GRADE)	Relative effect (95% CI)	Anticipated absolute effects	
				Risk of G6PDn patients	Risk of G6PDd patients (95% CI)
**Haemoglobin level (single low-dose PQ)**	397 patients in 4 studies	⨁⨁⨁◯ **Moderate** due to inconsistency and imprecision	–	The mean Hb level from **10.3 to 14.4** g/dL	The mean Hb level was **0.7 g/dL lower** (1.91 lower to 0.5 higher)
**Haemoglobin level (in low dose PQ)**	660 patients in 5 studies	⨁⨁⨁⨁ **High**	–	The mean Hb level from **10.3 to 14.4** g/dL	The mean Hb level was **0.04 g/dL lower** (0.35 lower to 0.27 higher)
**Anaemia in PQ therapy**	280 patients in 3 studies	⨁⨁⨁⨁ **High**	**RR = 1.02** (0.75 to 1.38)	**8 per 1000** patients	**8 per 1000** (from 97 fewer to 147 more)

*The risk in the G6PDd group (and its 95% CI) is based on the assumed risk in the G6PDn group

PQ: primaquine; CI: confidence interval

**GRADE Working Group grades of evidence**

**High quality:** We are very confident that the true effect lies close to that of the estimate of the effect

**Moderate quality:** We are moderately confident in the effect estimate: The true effect is likely to be close to the estimate of the effect, but there is a possibility that it is substantially different

**Low quality:** Our confidence in the effect estimate is limited: The true effect may be substantially different from the estimate of the effect

**Very low quality:** We have very little confidence in the effect estimate: The true effect is likely to be substantially different from the estimate of effect

There were several limitations in this study, i.e., heterogenous species of malaria parasites, various type and dose of administered drugs, small sample size in G6PD groups, and diverse method of outcomes measurement. Moreover, the G6PD status was not assessed genotypically, and phenotypes can be variable across different mutations. Higher quality clinical trials regarding the use of anti-malarial agents in G6PDd patients and their side effect on haemoglobin levels are recommended.

Based on this systematic review and meta-analysis, it was concluded that in G6PDd patients, the side effect of anaemia in single, daily low-dose (0.25 mg/kg/day), and weekly PQ was not statistically and clinically significant, and therefore, a massive screening of G6PD status might not be relevant in all populations. Several studies on the safety of anti-malarial regimens concerning G6PDd patients are ongoing (clinicaltrials.gov NCT03529396; NCT05044637; NCT05096702).

## Conclusion

Single low dose (0.25 mg/kg) or daily standard low dose of PQ (0.25 mg/kg/day) and weekly PQ (0.75 mg/kg/week for eight weeks) does not increase the risk of anaemia in G6PDd patients.

## Data Availability

Data generated and analysed in this study are included in this article. Additional data of the findings that are not included are available from the corresponding author, upon reasonable request**.**

## Abbreviations

CDA: Chlorproguanil–dapsone–artesunate
CI: Confidence interval
CPG–DDS: Chlorproguanil–dapsone
CQ: Chloroquine
G6PDd: Glucose-6-phosphate-dehydrogenase deficiency
G6PDn: Glucose-6-phosphate-dehydrogenase normal
GRADE: Grading of Recommendations Assessment, Development and Evaluation
MeSH: Medical Subject Headings
NOS: Newcastle–Ottawa scale
PQ: Primaquine
PRISMA: Preferred Reporting Items for Systematic Review and Meta-Analyses
PROSPERO: Prospective Register of Systematic Reviews
RCT: Randomized controlled trials
ROBINS: Risk of Bias In Non-randomized Studies–of Interventions
RR: Risk ratio
SD: Single dose
SE: Standard error
WHO: World Health Organization
